# Hyperbaric Oxygen Therapy for Children and Youth with Autism Spectrum Disorder: A Review

**DOI:** 10.3390/brainsci11070916

**Published:** 2021-07-11

**Authors:** Justyna Podgórska-Bednarz, Lidia Perenc

**Affiliations:** 1 Institute of Health Sciences, Medical College of Rzeszow University, Warzywna 1A, 35-310 Rzeszów, Poland; lidiaiadam.perenc@wp.pl; 2 Centre for Innovative Research in Medical and Natural Sciences, University of Rzeszow, Warzywna 1a, 35-310 Rzeszow, Poland

**Keywords:** autism, hyperbaric oxygen, therapy

## Abstract

Autism spectrum disorder (ASD) is a common neurodevelopmental disorder determined by a complex of factors (genetic and environmental). On a pathophysiological basis hyperbaric oxygen therapy (HBOT) has been suggested as an effective therapeutic method in ASD, and thus many parents/guardians attempt to treat their child with ASD using this method. Therefore, this review aimed to verify the significant therapeutic value of this method for individuals with ASD. The literature review included all articles from the last 5 years (2015–2021) that met the inclusion criteria—both original papers and literature reviews. None of the 10 literature reviews indicated that HBOT was a clearly effective form of therapy in the case of ASD. Two out of four papers presenting the results of the intervention studies also did not recommend the use of this form of therapy in children with ASD. The results of the other two studies were not entirely relevant to the purpose of this review because one study had no control group, while the other study focused solely on auditory processing disorders. A review of the literature on whether HBOT as a therapy significantly affects the symptoms of ASD does not confirm its effectiveness.

## 1. Introduction

Autism spectrum disorder (ASD) is a neurodevelopmental condition characterized by the following main groups of symptoms: social communicative and social interactive deficits; restricted and repetitive behaviors, interests or activities [1]. This developmental disorder can be diagnosed at any age, but generally its symptoms appear in early life period—before the age of three. ASD is a lifelong disorder, but proper therapies can improve a person’s functioning in every aspect of life. ASD is characterized by wide variation of symptoms and their severity and may appear in every racial, ethnic or economic population. Due to the relatively high prevalence, it is suggested that each child should be screened for ASD [2,3,4]. The latest data presented by the Centers for Disease Control and Prevention showed that the frequency of ASD diagnoses is increasing. In 2000, it amounted to 1 in 154 children, and the data from 2016 indicate a prevalence of about 1 in 54 children [5]. Moreover, its rising occurrence and often far-reaching influence on affected individuals and their families’ functioning present significant challenges for the public health and educational system [6].

As was mentioned before, people with ASD may encounter various problems, which significantly hinder their functioning in everyday life, both for themselves and their environment, such as: aggression, auto-aggression, irritability, hyperactivity, attention problems, depression and/or anxiety [7]. Due to the differentiation of symptoms, there is no single intervention model effective in the treatment of ASD. However, it underlines the fact that early diagnosis and implementation of therapy is a key factor in achieving optimal results—reducing difficulties, helping to learn new skills and developing strengths achieved through the use of highly structured and intensive behavioral, psychological and educational interventions, usually involving people from the immediate environment of the child—parents, teachers and other caregivers [2,8]. Despite the use of such intense interventions, sometimes there is a need to incorporate specific pharmacological treatment that can help in terms of behavior regulation, comorbid mood, impulse control and sleep disorders [8,9,10].

Unfortunately, even with the benefit of an intensive, comprehensive therapeutic process, people with ASD may often experience unfavorable repercussions of the disorder in the form of limited academic success, social rejection or distress, either directly affecting children/adults afflicted by the disorder or their caregivers or therapists [11,12]. For those reasons, parents/guardians very often look for other alternative treatments even if evidence for their benefits is lacking [13]. One of these methods is hyperbaric oxygen therapy (HBOT), which involves breathing high levels of oxygen inside a special chamber in which pressure is usually greater than 1.4 ATA (absolute atmosphere) [14]. Hyperbaric hyperoxia is an artificially induced phenomenon used with great success in HBOT in the therapy of late radiation-induced tissue damage, problematic wounds, decompression sickness or emboli [15]. On a pathophysiological basis (oxidative stress, cerebral hypoperfusion, inflammation, immune dysregulation and mitochondrial dysfunction) HBOT has been suggested as an effective therapeutic method also in ASD [16]. Cerebral hypoperfusion is phenomenon reported in persons with ASD in a number of studies [17]. The likely impact of HBOT therapy on improvement in terms of cerebral perfusion in individuals with ASD may also influence symptoms correlating with hypoperfusion that include decreased language development, repetitive behaviors, impairments in emotions and facial expressions and the desire for sameness [16]. Results of recent studies also suggest that a certain percentage of patients with ASD have neuroinflammation, immune dysregulation and/or gastrointestinal inflammation [18,19]. In this case, the use of HBOT therapy is based on its proven anti-inflammatory effect [16]. Another common physiological abnormality in this patient group is mitochondrial dysfunction [20]. An animal model demonstrated that HBOT led to increased mitochondrial biogenesis and autophagy—new healthy mitochondria are produced and old abnormal mitochondria are destroyed—which is partly due to the production of reactive oxygen species (ROS) [21]. It is also reported that a phenomenon of oxidative stress is observed in children with ASD [22]. HBOT increases levels of antioxidant enzymes and potentially protects against damage from ROS [23]. On the website of the centers offering HBOT therapy, parents/guardians can find information about a possible positive impact of HBOT therapy for this group of patients, which, however, has not been fully scientifically confirmed [24]. Therefore, despite the relatively high unit cost of the procedure, many parents decide to use this intervention expecting measurable positive benefits. However, it should be remembered that in addition to the risk associated with the lack of the expected therapeutic effects on symptoms connected with ASD, HBOT may also cause side effects in the form of ear barotrauma, fatigue, headaches or claustrophobia [25]. Despite the fact that HBOT is not a therapy recommended by specialists and institutions/organizations/expert teams related to the treatment of autism, many parents of afflicted children still take this form of therapy.

Therefore, this paper aimed to verify the significance of the therapeutic value of the method for individuals with ASD based on a review of recent literature.

## 2. Materials and Methods

**Figure 1:**
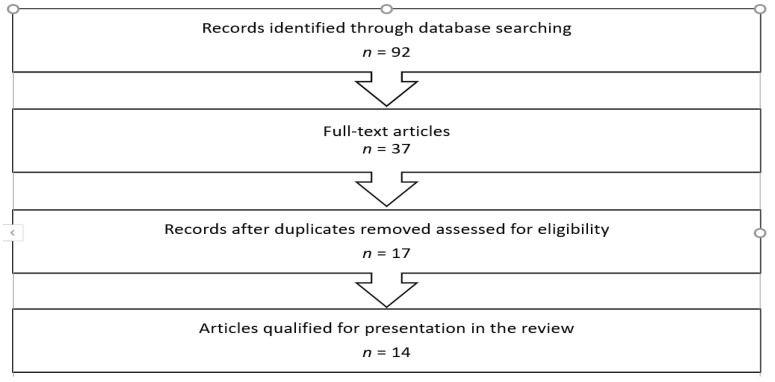
Literature selection process.

In order to find scientific articles relevant to the achievement of the aim of the study, electronic searches were used. For this purpose, the following databases were used: PubMed, EBSCO, ScienceDirect, Ovid, Science, Springer, Scopus, Cochrane Database, Web of Science, Wiley Online Library. The literature review included all articles from the last 5 years (2015–2021; articles published until March 2021 were also analyzed) that met the inclusion criteria. The following keywords were used: autism, autistic, autism spectrum disorder, ASD, hyperbaric oxygen therapy, hyperbaric oxygen, HBOT. The criteria for inclusion in the review were as follows: (1) all types of articles/research published in the analyzed period, (2) articles in all languages with abstracts in English, (3) research/review presented using HBOT therapy in children and/or adolescents—age range up to 19 years of age, (4) main subject of article: HBOT/CAM and ASD. The total number of retrieved records was 92 with 37 full-text papers; after elimination of duplicates and ineligible articles, the final number of reports discussed in the paper was 14. A flow chart presenting the detailed process of literature selection is presented in Figure 1. Then, the selected articles were systematized into articles presenting literature reviews and research results (Table 1a,b).

## 3. Results

As previously mentioned, the final result of the literature analysis consisted of 14 articles, including 10 reviews (Table 1a) and 4 original papers (Table 1b). With regard to the review papers, 1 analysis from 2020, 2 from 2017, 3 from 2016 and 4 from 2015 were cited in the paper. Zhukova et al. in their report, dedicated as a review to parents and clinicians, divided complementary and alternative treatments (CAT) into different subgroups depending on their effectiveness, classifying HBOT as “harmful, inconclusive or no efficacy” method for patients with autism [26]. However, they made this conclusion only on the basis of a systematic review by Xiong et al. based on RCT (randomized controlled trials) and quasi-RCT, results of which were published before 10 December 2015. The authors of the article not only observed the positive results of the therapy but also questioned the RCT offer of HBOT therapy in this group of patients on the basis of the analyses of previous studies. [29]. In addition, in the overview of systematic reviews from 2017, HBOT was classified as an intervention that did not bring the expected therapeutic results [28]. Authors of the remaining reports present similar conclusions [27,30,31,32,34,35] apart from one, classifying HBOT as a potentially promising therapy based on the pathophysiology of ASD [33]—main outcomes from the articles are presented in Table 1a.

The second distinguished group were intervention studies from the last 5 years—4 studies from the following years: 2021, 2020, 2019 and 2018. Interesting results were presented by Kostiukow and Samborski on the improvement of various specific functions (divided into younger and older groups) determined with the use of tools typical for the assessment of ASD. Although the results were based on a relatively large study group, the research did not include a control group. Therefore, it is not possible to unequivocally assess the results obtained by Kostiukow as supporting the use of HBOT therapy in children with ASD [37]. In turn, in a study on the effect of HBOT on both core symptoms and pathophysiology of ASD, no significant improvement was found when compared with the control groups [36]. Promising results on auditory processing disorders in children with ASD were presented by Lasheen and his research team, who noted an improvement in both auditory memory and auditory attention in the study group compared with the control group after the HBOT sessions [38].

The original papers selected for review essentially assessed various aspects of abnormalities that may occur in children and adolescents with ASD. The results of the presented research can be divided into those obtained through the use of psychometric tools and biological measurements.

In one of the original papers aimed at verifying possible changes in selected psychometric parameters, three psychometric tools were used: (1) Clinical Global Impression Scale (CGI), consisting of a 7-point symptom severity scale and a 7-point overall improvement scale; (2) Autism Treatment Evaluation Checklist (ATEC), consisting of 4 subscales evaluating speech/language/communication, sociability, sensory/cognitive awareness and health/physical/behavior and (3) Childhood Autism Rating Scale (CARS), which allows the assessment of the following aspects: relationship to people, imitation, emotional response, body use, object use, adaptation to change, visual response, listening response, taste-smell-touch response and use, fear and nervousness, verbal communication, non-verbal communication, activity level, level and consistency of intellectual response and general impression. Referring to the statistically significant results of the ATEC checklist, the following were obtained: (1) declined after HBOT therapy in item speech/language/communication—“Can follow some commands” for the whole group; (2) significant improvement in items: (2a) for the whole group: Sensory/cognitive awareness—“Shows imagination”, Health/physical/behavior—“Sound-sensitive”, Health/physical/behavior—“Obsessive speech”, (2b) for the younger group (under 79 months): sociability—“Does not imitate”, sensory/cognitive awareness—“Shows imagination”, Health/physical/behavior—“Sound-sensitive” and (2c) for the older group (over 79 months), improvement in the scope of one parameter: health/physical/behavior—“Obsessive speech”. The CGI results showed a significant improvement in the symptom severity subscale, but it only referred to the older age category. In terms of the study, the CARS tool identified an improvement in six aspects for the entire study group (imitation, emotional response, object use, adaptation to change, general impression, total score) and three aspects relating to the older age group (emotional response, adaptation to change and total score). Unfortunately, the study also had some significant limitations, such as the lack of a control group [37] (Table 1b). In another research project, two tools were used to analyze the psychometric aspect: the previously described CARS and the Aberrant Behavior Checklist (ABC), which comprises a total of 58 items and is based on observation of the following categories: sensory responses (ABC-1), affective responses (ABC-2), stereotypies and use of objects (ABC-3), language development (ABC-4) and individual and social autonomies (ABC-5)—lower scores indicate less severe ABC autism. The total results of ABC measurements taken before and one month after HBOT therapy show a statistically significant improvement in both groups: study and control. When analyzing the subscales, no significant improvement was observed for: ABC-2, ABC-3 and ABC-5 in the study group. CARS results were not statistically significant. In addition, the study was conducted under conditions of nutritional standardization aimed at minimizing proinflammatory sources of nutrition [39] (Table 1b). Interesting research results are presented in the work combining biological measurements with psychometric analysis. The research results indicated that ASD-related behavioral outcomes moderately correlate with platelets’ mitochondrial bioenergetic parameters. They also are well linked with NOX-mediated activity in neutrophils. HBOT was not shown either to improve mitochondrial dysfunctions or to counteract ASD-related behavioral deficits. Although HBOT improved one measure of the immune response—namely, via a NOX-mediated superoxide burst—this was not associated with significant changes in trends of recurrent infections between the groups [36] (Table 1b). The final cited interventional study was based on the measurement of specific biological functions of the body—comparing P300 and MMN latencies and amplitudes of ASD patients before and after HBOT treatment. The test results showed a significant decrease in P300 and MMN latencies and admittedly increased the amplitude of P300 and MMN, but the result was not statistically significant [38] (Table 1b).

In order to summarize the literature, it should be remembered that only one review article states the fact of a positive potential (“promising results”) [33] effect of HBOT therapy on patients with ASD. Referring to interventional studies, also only one of them clearly indicates the potential effects of this therapy; however, this applies not to the assessment of core symptoms but only to improvement in auditory attention and auditory memory [38]. All the remaining research indicates a potentially low benefit or a lack of benefit from the therapy in relation to the expenditure and the potential risk associated with it.

## 4. Discussion

Due to the frequency of occurrence and the specificity of the disorder, ASD has become an important subject of research and public interest for a long time. Reports on new methods potentially effective in “treating” autism appear with a relatively high frequency in both the professional literature and in the daily press. One of the methods in which parents/guardians place great hopes is HBOT therapy due to the pathophysiology that characterizes ASD—which operates causally rather than symptomatically. The problem in the case of using HBOT as CAM (complementary and alternative medicine) therapy is its relatively high cost and time consumption, with effectiveness not fully confirmed and with possible side effects.

Of course, HBOT therapy is defined as recommended and effective for many conditions, but it should be remembered that in connection with its use, there may also occur side effects such as middle ear barotrauma (MEB), sinus/paranasal barotrauma, dental barotrauma, central nervous system (CNS) oxygen toxicity, pulmonary oxygen toxicity, hyperoxic myopia, cataracts, retrolental fibroplasia following hyperoxic exposure, claustrophobia or increase in blood pressure, pulmonary edema and hypoglycemia in diabetics [40]. Therefore, basically due to the mechanism, the side effects can be divided into physiological reactions related to the use of high pressure and high oxygen concentration, as well as psychological reactions related to being in the confined space of a chamber [40,41]. An interesting example is MEB, one of the most common side effects of HBOT therapy, characterized by a feeling of pressure, discomfort and pain during the first phase of treatment. MEB can lead to edema in the middle ear, retraction, rupture of the tympanic membrane and rupture of the round or oval window membranes, and the consequences of these failures include conductive hearing deficits and/or vertigo and sensorineural hearing loss [42]. The mechanism of this pathology is based on relative negative middle ear pressure causing a chain of irregularities in the form of collapse and closure of the eustachian tube (ET), contraction of gas volume in case of inability to open the ET, inward retraction of the tympanic membrane and adjoining ossicles, middle ear mucosal swelling, capillary dilation, transudate leakage, fluid extravasation into the middle ear space, blood vessel rupture, hemotympanum and possible tympanic membrane perforation [43]. Another important issue covered by numerous studies is CNS oxygen toxicity, caused by increased partial pressure of oxygen that saturates protective enzymes, which can lead to overstimulation of the neural network [44]. On the other hand, claustrophobia (defined as fear of being enclosed in small spaces) is an example of the psychological side effects that may result from HBOT therapy, which may have a basis both in past experience and conditions related to the specific structure of the CNS (smaller amygdala) [45]. It should also be remembered that the stress associated with conducting this type of therapy, consisting in temporary confinement of a patient with ASD in a limited space and potential reactions that could lead to injuries throughout the procedure.

Unfortunately, none of the presented articles provide strong evidence of the legitimacy of the use of hyperbaric oxygen therapy for autism. Therefore, clinicians cannot recommend it as a safe and scientifically confirmed, effective method. Until the results of the research are unequivocal, in which case HBOT should be used in groups of patients with developmental disorders, parents/guardians need to be informed that at the moment this is not recommended as an effective form of therapy for this disorder. Additionally, following the current reports, parents would be better advised to expend their resources on forms of treatment with clearly proven effectiveness [46]. Moreover, as previously mentioned, HBOT therapy is not a therapy, which in the case of a diagnosis of ASD is a service reimbursed by the health care system or insurance institutions. Due to the need for a fairly large number of sessions and its high unit cost, it poses a serious challenge for the home budget of parents/caregivers. It should also be remembered that currently it is not a form of therapy with scientifically proven effectiveness in the case of ASD; it is only one of the methods of alternative therapy. The research methodology should also be carefully considered, which is significantly different, often without specifying whether other forms of therapy were abandoned during the application of HBOT therapy. It should also be taken into account that, to the best of the authors’ knowledge, there are no reports in the literature regarding the duration of the therapeutic effect in the case of HBOT therapy—associated with the possibility of a relatively quick return to the pre-therapy state after its discontinuation, as well as with possible long-term negative consequences. One should also remember the previously discussed possible side effects of the therapy, as well as the stress associated with placing a child/teenager in a closed space such as the chamber.

It should also be noted that this article has some limitations. The review was not conducted according to the PRISMA guidelines; it was limited to the period 2015–2021. Gray literature sources were not taken into account. We did not assess the strength of the body of evidence. Another limitation of the work was the focus only on the developmental population; we did not take into account the research on adults.

## 5. Conclusions

A review of the literature on whether HBOT as a therapy significantly affects the symptoms of ASD does not confirm unequivocally its effectiveness. HBOT is therefore not a recommended form of therapy in the case of ASD. It is possible that future research will reveal specific groups of children for whom the use of HBOT could be beneficial due to their specific problems, but the current state of knowledge does not confirm the legitimacy of its use in the entire developmental population diagnosed with ASD.

## Figures and Tables

**Table 1:** Systematics of reports cited in the review.

**(1a) Reviews**	**Number of** **Patients/** **Participants/** **Cases**	**Methodology**	**Main Outcome**	**Measures**
Zhukova, M.A. et al. (2020) [26]	N/A	N/A	“We advise against the use of (…) hyperbaric oxygen therapy, (…) due their documented harmful psychological and physical effects.”	N/A
Sakulchit, T. et al. (2017) [27]	N/A	N/A	“Currently, there is insufficient evidence to support use of HBOT to treat children with ASD (…)”	N/A
Lyra, L. et al. (2017) [28]	N/A	N/A	“No benefits were found for (…) hyperbaric oxygen therapy (…)”	N/A
Xiong, T. et al. (2016) [29]	N/A	N/A	“To date, there is no evidence that hyperbaric oxygen therapy improves core symptoms and associated symptoms of ASD.”	N/A
Klein, N. et al. (2016) [30]	N/A	N/A	“Given their risks, costs, and limited evidence of efficacy, chelation, secretin, and hyperbaric oxygen should be avoided.”	N/A
Goldfarb, C. et al. (2016) [31]	N/A	N/A	“Current evidence does not support HBOT as an effective treatment for children and youth with ASD.”	N/A
Martin, R. et al. (2015) [32]	N/A	N/A	“The evidence is weak for the use of HBO2 in ASD, with only one, likely flawed, randomized control study showing treatment benefit.”	N/A
Li, HH. et al. (2015) [33]	N/A	N/A	“With the in-depth study of the pathogenesis of ASD, bumetanide, oxytocin, vitamin D and hyperbaric oxygen therapy have been found to be promising for the improvement of core symptoms of ASD.”	N/A
Brondino, N. et al. (2015) [34]	N/A	N/A	“In conclusion, there are still few data on the potential efficacy of CAM in autism, and no evidence-based recommendation could be done so far for the use of such therapies.”	N/A
Politte L.C. et al. (2015) [35]	N/A	N/A	“At this time, the use of HBOT for the treatment of ASD is not recommended.”	N/A
**(1b) Intervention Studies**	**Number of** **Patients/** **Participants/** **Cases/Age Range**	**Methodology**	**Main Outcome/Side Effects—if Any**	**Measures**
Abdel-Rahman, E.A. et al. (2021) [36]	Study group: *n* = 10 children with ASD, 5.47 ± 0.87 years; I Control group: n = 10 neurotypical children, 5.28 ± 0.75 years II Control group: *n* = 10 children with autism + no HBOT treatment, 4.75 ± 0.72 years	HBOT sessions number = 10—75; 37.6 ± 12.2	“We also found no evidence that HBOT confers any significant improvement of ASD-associated physiological or behavioural phenotypes.” “Similarly, no detectable improvement in ASD-associated behavioral deficits in HBOT group relative to untreated autistic group.”/not reported	Childhood Autism Rating Scale (CARS), Autism Treatment Evaluation Checklist (ATEC), Vineland Adaptive Behavior Scales (VABS), high resolution respirometry, activity of nicotinamide adenine dinucleotide phosphate (NADPH) oxidase (NOX) in isolated neutrophils
Kostiukow, A. et al. (2020) [37]	Study group: *n* = 35 boys mean age of 6.9 years, min/max = 2.0/15.8 ± 3.0; 4 girls with ASD mean age 10.2 years, min/max = 4.7/16.0 ± 4	HBOT sessions number = 40; 1.5 atm; 60 min/daily/8 weeks or 4 weeks when 2 sessions a day	“(…) ATEC Speech/language/communication—“Can follow some commands” revealed a decline (…)” “Eight components of the ATEC and CARS scales as well as the CARS total score revealed statistically significant improvements.”/not reported	Clinical Global Impression Scale (CGIS), Autism Treatment Evaluation Checklist (ATEC) and Childhood Autism Rating Scale (CARS)
Lasheen, R.H. et al. (2019) [38]	Study group: *n* = 20 children with ASD, Age =10.6 ± 2.4; Control group: *n* = 20 children (neurotypical) chronologically age-matched	HBOT sessions number = 40; 1.5 ATM; 45 min/day/total of 40 sessions	“The children with autism showed improvement in both auditory attention and auditory memory after hyperbaric oxygen therapy.”/not reported	Auditory P300 and MMN (Mismatch Negativity)
Rizzato, A. et al. (2018) [39]	Study group: *n* = 7 boys: 1 girl with ASDF = 1, mean age: 7 ± 2.33; years; Control group: *n* = 5 boys, 2 girls (with ASD), 6.6 ± 2.7 years	HBOT sessions number = 40; 8 weeks	“Despite the improvements reported in both groups, our results do not support the utility of HBO2 in children diagnosed with autism.”/not reported	Aberrant Behavior Checklist-Community (ABC), Childhood Autism Rating Scale at T0 and T2

N/A—not applicable.
